# Lifestyle and Environment Influence the Psychological Well-Being of Elderly Subjects in Italy

**DOI:** 10.3390/brainsci14121276

**Published:** 2024-12-19

**Authors:** Simone Migliore, Marco De Angelis, Ilaria Di Pompeo, Daniele Lozzi, Martina Marcaccio, Giuseppe Curcio

**Affiliations:** 1 Department of Biotechnological and Applied Clinical Sciences, University of L’Aquila, 67100 L’Aquila, Italy; simone.migliore@univaq.it (S.M.); marco.deangelis@univaq.it (M.D.A.);; 2 Department of Life, Health and Environmental Sciences, University of L’Aquila, 67100 L’Aquila, Italy; daniele.lozzi@graduate.univaq.it

**Keywords:** active aging, physical activity, cognitive functions, elderly, psychological health

## Abstract

Background/Objective: Aging is associated with both cognitive and physical decline. Some factors, such as lifestyle and environment, can significantly contribute to accelerating or slowing down the decline processes. Our study aimed to evaluate the impact of lifestyle (active vs. non-active) and environmental context (institutionalized vs. non-institutionalized) on the cognitive functioning, psychological well-being, sleep quality, and daily living skills of elderly people. Methods: Our sample consisted of 182 subjects divided into active and non-active groups (subjects who engage or not in physical and social activities, respectively; mean age in years: 67.19 vs. 68.75) and 245 subjects divided into institutionalized and non-institutionalized groups (i.e., living in a nursing home or not, respectively; mean age in years: 79.49 vs. 71.72). Participants were enrolled voluntarily and randomly in the city of L’Aquila. A battery of psychological instruments was administered to evaluate general cognitive decline, depressive symptoms, self-assessed sleep quality, and daily living skills. Results: Regarding lifestyle, the active group exhibited significantly lower levels of depression, better sleep quality, and daily living skills with respect to the non-active group. Regarding environmental context, institutionalized subjects showed higher levels of depression and reduced cognitive functioning, which were linked to reduced sleep quality and worsened daily living skills. When comparing the non-active with the institutionalized group, the latter showed higher levels of depression and reduced cognitive functioning, more sleep complaints, and reduced daily living skills. Conclusions: Our study highlights that an active lifestyle and a non-institutionalized environment, both allowing greater mobility and autonomy, are two factors that positively contribute to the mental and physical well-being of elderly individuals. Furthermore, the healthcare institution context appears to have a greater negative impact on the psycho-physical well-being of the subjects involved compared to a non-active lifestyle.

## 1. Introduction

Over the past few decades, we have witnessed significant shifts in the age demographics of human populations, particularly in industrialized nations. These shifts are marked by a rising likelihood of individuals attaining old age and extreme longevity [1,2,3]. Aging, being a multifaceted aspect of life, is a challenging phenomenon to define in simple terms [4]. The World Health Organization describes aging as a “process of progressive change in the biological, psychological, and social structures of individuals” [5].

From a biological perspective, aging is often equated with senescence, described as “a biological process involving dysfunctional changes that reduce an organism’s ability to maintain physiological functions and homeostasis as survival increases” [6]. These and other definitions highlight the complexity of a precise definition of the phenomenon of aging [4]. Generally, aging is associated with a progressive decline in perception, cognition, and memory [7], along with a deterioration in physiological capacities such as muscle strength, aerobic capacity, and neuromotor coordination [8]. Although these changes can vary greatly, there is a high likelihood that older adults will experience age-related dysfunctions [9], which challenge their ability to maintain independence in daily life. These increased dysfunctions underscore the importance of better understanding the mechanisms of the human aging processes and developing strategies to preserve health and functional independence.

Independence in daily activities is considered a key element of successful aging (SA), which is defined by the absence of major diseases and disabilities, the maintenance of high levels of physical and cognitive function, and the continuation of social and productive activities [10,11]. In this context, SA acts as a protective factor against neurodegenerative processes, representing a complex strategy whereby the brain attempts to withstand damage resulting from physiological or pathological aging. This is achieved through brain plasticity processes and the reorganization of cognitive functions [12,13,14]. Moreover, SA extends beyond merely avoiding disease and disability, focusing instead on achieving a high level of functioning and satisfaction in various life domains (i.e., physical, psychological, and social) [15]. The idea is to create a positive, active, and fulfilling life as one grows older. Because of reduced functional abilities and limited energy reserves, older adults are more susceptible to challenges posed by their physical and social surroundings compared to younger individuals [16]. Designing and planning communities that encourage activity across all age groups should be based on a thorough understanding of the intricate interactions between individuals and their environments, taking into account the interconnected physical, social, and technological elements. A recent meta-analysis, for example, indicated that age-friendly environments may be more effective in promoting the emotions of older adults, compared to ameliorating their physical functioning [17].

Recent research indicates that the benefits of independence in daily life activities and physical exercise in old age extend beyond physical advantages to include psychological and cognitive improvements as well [18,19,20]. Regular physical activity, in conjunction with mental exercises and social engagement, serves as a crucial protective factor for SA. A recent study suggests that a lifestyle including physical activity may contribute to supporting daily cognitive function for older adults, in particular memory functioning [21]. Types of physical activity, as well as the intensity and frequency with which they are practiced, are factors that protect the psychophysical well-being and overall cognitive functioning of the elderly [21,22], with improvements also in flexibility, balance, and coordination [23], which lead to a decreased risk of falls [24] and enhancements in movement speed and accuracy [25,26]. As age advances, along with associated physical and cognitive changes, and with increasingly smaller family units resulting in a reduced capacity for assistance and support, there is often a need to resort to specialized institutions for the care and support of elderly individuals.

Institutionalization and a sedentary lifestyle can have significant negative repercussions on both physical and cognitive levels. These factors often exacerbate each other, leading to a decline in overall health and quality of life [27]. For example, from a physical perspective, they could exhibit muscle atrophy and loss of strength, cardiovascular health decline, obesity and metabolic disorders, decreased immune function, and respiratory issues, and much more could also arise [28]. From a cognitive and psychological perspective, they could exhibit an accelerated cognitive decline, depression and anxiety, apathy, lower brain plasticity, and poor quality of life [29,30].

Our study aimed to evaluate the impact of lifestyle (active vs. non-active) and environmental context (institutionalization vs. non-institutionalization) on older subjects in terms of their cognitive functioning, psychological well-being, sleep quality, and daily living skills. To our knowledge, this has not been attempted already in the Italian population and, if attempted, never through a relevant sample size.

## 2. Materials and Methods

### 2.1. Participants

All the participants voluntarily participated in this study and signed an informed consent form; the study protocol was conducted in accordance with the Declaration of Helsinki and approved by the Internal Review Board (#22/2017). Participants were recruited voluntarily and randomly from the general population in the city of L’Aquila. The questionnaires were administered at the University of L’Aquila, Department of Biotechnological and Applied Clinical Sciences, Cognitive and Behavioral Sciences Laboratory (LabSCoC), from January to June 2024, by two expert psychologists (S.M. and G.C.). Institutionalized participants were recruited randomly and voluntarily thanks to the collaboration of three separate nursing homes in the area surrounding the city; each of them agreed to participate in the project. In this case, the questionnaires were administered at the nursing home by the same two expert psychologists.

### 2.2. Active vs. Non-Active

A total of 182 participants (mean age in years: 67.87 ± 6.21) were enrolled. The subjects were categorized as “active” (Ac) if they met at least 3 out of the following 5 criteria: performed gym or sporting activities (such as tennis, golf, jogging, or gymnastics exercise) at least once a week; practiced hobbies (such as DIY activities, gardening, or collecting) at least once a week; read newspapers (online or in print) at least three times a week; attended the cinema or theater at least once a month; and participated in social activities (such as church, volunteering, or political parties) at least once a month. Conversely, subjects who did not meet at least 3 of the 5 previously mentioned criteria were classified as “non-active” (NAc). General cognitive decline was screened through the Mini-Mental State Examination (MMSE), and participants with a score of less than 22/30 were excluded to avoid early stages of dementia and potential confounding effects on the interpretation of the results.

### 2.3. Institutionalized vs. Non-Institutionalized

A total of 245 participants (mean age in years: 74.61 ± 7.87) were enrolled. In this case, subjects were categorized as “institutionalized” (Is) if they lived in a healthcare facility for at least 3 months and no more than 1 year. We considered a one-year time limit because individuals with longer institutionalization periods are typically characterized by multiple comorbidities, both physical and cognitive–behavioral. These factors could negatively impact and potentially confound the interpretation of the results. Participants were categorized as “non-institutionalized” (NIs) if they lived in their own homes (either alone or with their partner). Subjects in the non-institutionalized group met the criteria to be classified as inactive because they did not meet at least 3 of the 5 criteria identified to be classified as active. The participants in both the Is and NIs groups were required not to have motor impairments that would prevent independent walking or autonomy in performing basic activities of daily living. Moreover, general cognitive decline was screened through the MMSE, and participants with a score of less than 22/30 were excluded to avoid early stages of dementia and potential confounding effects on the interpretation of the results.

### 2.4. Instruments

Participants’ performances in terms of cognitive abilities, psychological well-being, sleep quality, and daily living skills were rated with a number of standardized instruments that included the MMSE [31], Geriatric Depression Scale—short form (GDS) [32], Pittsburgh Sleep Quality Index (PSQI) [33], and Everyday Competence Questionnaire (ECQ) [34].

The cognitive evaluation aimed to provide a measure of general mental efficiency. To this end, the MMSE is a widely used clinical tool designed to assess cognitive function and screen for cognitive impairments, such as those associated with dementia: registration (i.e., repeating named prompts), attention and calculation, recall, language, ability to follow simple commands, and place/time orientation. The MMSE remains one of the most commonly used cognitive screening instruments. The MMSE is scored out of a total of 30 points, where higher scores indicate better cognitive functioning.

The GDS is a patient-reported outcome measure to screen for depressive symptoms among older adults: depression involves a depressed mood or loss of pleasure or interest in activities for long periods of time. The GDS is a multidimensional, multidisciplinary diagnostic and therapeutic activity that helps find subjects at risk of/with depression. Each yes/no response is assigned a point. Scores are summed to indicate the level of depressive symptoms.

The PSQI is a short self-reported questionnaire for the evaluation of sleep quality referring to the last four weeks. The PSQI provides a total score and 7 composite indices: subjective sleep quality, C1 (how participants rate—good/bad—their usual sleep); sleep latency, C2 (how long participants rate their usual period of falling asleep); sleep duration, C3 (how participants rate their usual sleep length); habitual sleep efficiency, C4 (how much the participants sleeping time and time in bed coincide); sleep disturbances, C5 (how much participants complain about sleep troubles); use of sleep medications, C6 (how much participants report use of medications to improve sleep); and daytime dysfunction, C7 (how participants complain about daytime difficulties). Higher scores indicate greater impairment of sleep quality as well as of specific components.

The ECQ is a tool designed to measure an individual’s ability to perform everyday tasks and activities independently, particularly in older adults. Higher scores indicate greater competence and independence. The ECQ provides a total score (ECQ_tot, indicating the general independence in everyday life) and 8 subscores: housekeeping (ECQ_hk, indicating how independent participants are in housekeeping activities), leisure activities (ECQ_lsa, indicating how engaged participants are in hobbies and pleasant activities such as gardening, playing a musical instrument, etc.), sports (ECQ_s, indicating how engaged participants are in sporting activities), daily routines (ECQ_dr, indicating how involved participants are in daily activities such as shopping, cooking, etc.), manual skills (ECQ_ms, indicating how able participants are in computer writing, housework, etc.), subjective well-being (ECQ_swb, indicating how participants rate their general health), mobility (ECQ_m, indicating how much participants travel and/or use bicycles), and general linguistic usage (ECQ_la, indicating the fluency and efficiency of participants’ speech).

### 2.5. Statistical Analyses

The demographic data were reported as means and standard deviations (SD). The chi-square test and Student’s *t*-test were conducted to assess the statistical difference between group characteristics. Statistical analyses were conducted by comparing the groups pairwise (Ac vs. NAc; Is vs. NIs; NAc vs. Is). Regarding clinical scale (MMSE, GDS, and PSQI) and individual ability (ECQ), all dependent variables were submitted to a one-way analysis of variance (ANOVA), directly comparing the performances of different groups. Also, analyses of covariance (ANCOVAs) were performed to evaluate the differences observed between groups, adjusting for demographic variables (such as age, education, and gender), where statistically significant differences were observed between groups.

A *p*-value < 0.05 was considered significant. All statistical analyses were performed using Jamovi (version 2.6.19).

## 3. Results

### 3.1. Active (Ac) vs. Non-Active (NAc)

Student’s *t*-test revealed statistically significant differences in educational level (*p* = 0.012) between the Ac (10.8 ± 4.56) and NAc groups (9.03 ± 4.81). The differences in demographic variables are reported in Table 1.

**Figure 1:**
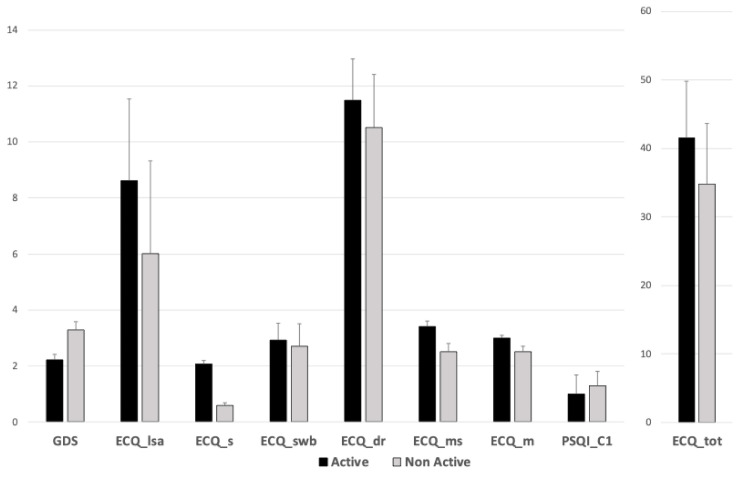
Statistically significant differences in the active and non-active groups’ scores. GDS: Geriatric Depression Scale; ECQ_lsa: leisure activities; ECQ_s: sports, ECQ_swb: subjective well-being; ECQ_dr: daily routines; ECQ_ms: manual skills; ECQ_m: mobility; PSQI_C1: subjective sleep quality; ECQ_tot: Everyday Competence Questionnaire, total score.

The one-way ANOVA revealed several significant group effects. Specifically, the NAc group showed significantly higher scores compared to the Ac group in the depression scale (GDS—F_1,158_ = 8.214; *p* = 0.005; η^2^ = 0.046; 95% CI: 2.33–3.05), while the Ac group showed significantly higher scores in daily living skills and specifically in leisure activities (ECQ_lsa—F_1,159_ = 29.9; *p* < 0.001; η^2^ > 0.14; 95% CI: 6.99–7.98), sports (ECQ_s F_1,179_ = 79.627; *p* < 0.001; η^2^ > 0.14: 95% CI: 1.24–1.64), subjective well-being (ECQ_swb—F_1,149_ = 4.352; *p* = 0.039; η^2^ = 0.025; 95% CI: 2.74–2.95), daily routine (ECQ_dr—F_1,148_ = 6.316; *p* = 0.013; η^2^ = 0.102; 95% CI: 10.93–11.44), manual skills (ECQ_ms—F_1,168_ = 6.887; *p* = 0.009; η^2^ = 0.037; 95% CI: 2.69–3.36), mobility (ECQ_m—F_1,153_ = 8.795; *p* = 0.004; η^2^ = 0.048; 95% CI: 2.42–2.93), and ECQ total score (ECQ_tot—F_1,163_ = 26.979; *p* < 0.001; η^2^ = 0.132; 95% CI: 37.20–39.89). Finally, regarding the sleep self-assessment, the NAc group showed significant higher scores in the subjective sleep quality subscore (PSQI_C1—F_1,93_ = 5.862; *p* = 0.017; η^2^ = 0.051; 95% CI: 0.99–1.25). Such statistically significant scores are depicted in Figure 1. Moreover, no statistically significant differences between the groups were seen for other ECQ (ECQ_la *p* = 0.647; ECQ_hk *p* = 0.616) or PSQI subscores (PSQI_C2 *p* = 0.416; PSQI_C3 *p* = 0.576; PSQI_C4 *p* = 0.612; PSQI_C5 *p* = 0.471; PSQI_C6 *p* = 0.561; PSQI_C7 *p* = 0.91; PSQI_tot *p* = 0.737), as well as for MMSE scores (*p* = 0.182).

Finally, an ANCOVA was conducted to assess the effects of demographic variables (age, education, and gender) on the variables of interest previously mentioned, revealing no significant effects for any of the demographic factors.

### 3.2. Institutionalized (Is) vs. Non-Institutionalized (NIs)

Student’s *t*-test revealed statistically significant differences in age (*p* < 0.001) between the Is (79.49 ± 7.03) and NIs groups (71.72 ± 6.86). Differences in demographic variables are reported in Table 2.

**Figure 2:**
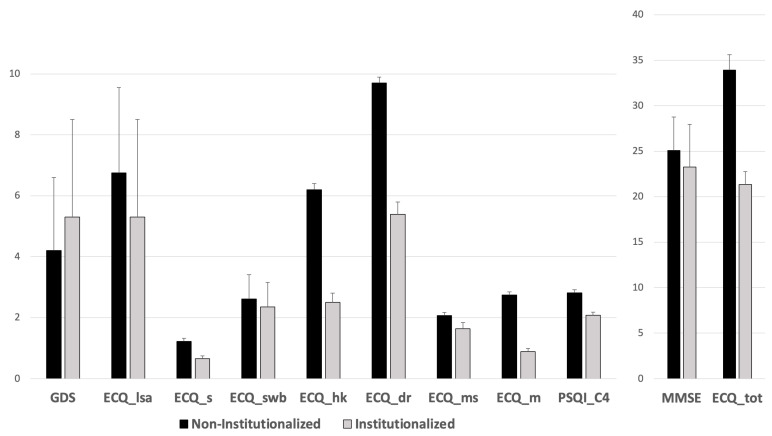
Statistically significant differences in institutionalized and non-institutionalized groups. GDS: Geriatric Depression Scale; ECQ_lsa: leisure activities; ECQ_s: sports, ECQ_swb: subjective well-being; ECQ_hk: housekeeping; ECQ_dr: daily routines; ECQ_ms: manual skills; ECQ_m: mobility; PSQI_C4: habitual sleep efficiency; MMSE: Mini-Mental State Examination; ECQ_tot: Everyday Competence Questionnaire, total score.

One-way ANOVA revealed several significant group effects. Specifically, the Is group showed significantly higher scores compared to the NIs group in depression scale (GDS—F_1,164_ = 6.743; *p* = 0.01; η^2^ = 0.036; 95% CI: 4.32–5.17) and significantly lower scores in general cognitive functioning (MMSE—F_1,154_ = 9.701; *p* = 0.002; η^2^ = 0.043; 95% CI: 23.86–24.93). Moreover, the Is group showed significantly lower scores in daily living skills and specifically in leisure activities (ECQ_lsa—F_1,168_ = 12.632; *p* < 0.001; η^2^ = 0.053; 95% CI: 5.83–6.60), sports (ECQ_s F_1,207_ = 20.353; *p* < 0.001; η^2^ = 0.073; 95% CI: 0.89–1.14), subjective well-being (ECQ_swb—F_1,188_ = 6.198; *p* = 0.014; η^2^ = 0.025; 95% CI: 2.42–2.62), housekeeping (ECQ_hk—F_1,132_ = 99.243; *p* < 0.001; η^2^ > 0.14; 95% CI: 4.45–5.22), daily routine (ECQ_dr—F_1,130_ = 75.342; *p* < 0.001; η^2^ > 0.14; 95% CI: 7.61–8.70), manual skills (ECQ_ms—F_1,198_ = 3.888; *p* = 0.05; η^2^ = 0.015; 95% CI: 1.70–2.13), mobility (ECQ_m—F_1,229_ = 85.157; *p* < 0.001; η^2^ > 0.14; 95% CI: 1.82–2.29), and ECQ total score (ECQ_tot—F_1,146_ = 61.763; *p* < 0.001; η^2^ > 0.14; 95% CI: 27.65–30.85). Furthermore, regarding sleep quality and related complaints, the Is group showed significantly higher scores in subjective sleep quality subscore (PSQI_C4—F_1,123_ = 23.975; *p* < 0.001; η^2^ = 0.115; 95% CI: 2.41–2.67), the use of sleep medications (PSQI_C6—F_1,162_ = 4.143; *p* = 0.043; η^2^ = 0.018; 95% CI: 0.50–0.78), and daytime dysfunction (PSQI_C7—F_1,188_ = 4.261; *p* = 0.04; η^2^ = 0.017; 95% CI: 0.65–0.84). Such statistically significant scores are depicted in Figure 2. No statistically significant differences between the groups were seen for other ECQ_la subscore (*p* = 0.958) or PSQI subscores (PSQI_C1 *p* = 0.978; PSQI_C2 *p* = 0.46; PSQI_C3 *p* = 0.111; PSQI_C5 *p* = 0.066; PSQI_tot *p* = 0.066).

Finally, an ANCOVA was conducted to assess the effects of demographic variables (age, education, and gender) on the variables of interest previously mentioned, and the analysis revealed no significant effects for any of the demographic factors. 

### 3.3. Institutionalized (Is) vs. Non-Active (NAc)

Student’s *t*-test revealed statistically significant differences in age (*p* < 0.001) between the Is (79.49 ± 7.03) and NAc groups (68.75 ± 6.14). Differences in demographic variables are reported in Table 3.

**Figure 3:**
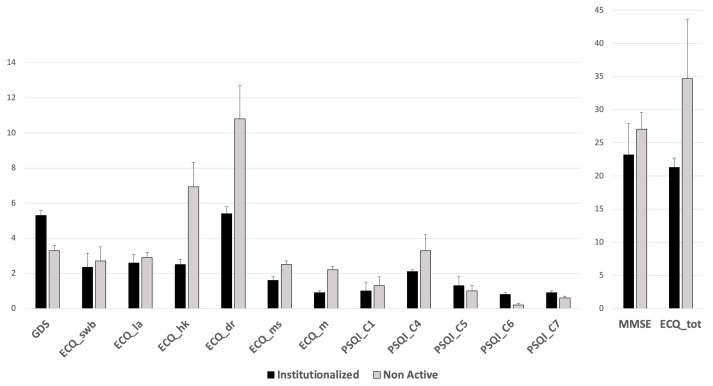
Statistically significant differences in institutionalized and non-active groups. GDS: Geriatric Depression Scale; ECQ_swb: subjective well-being; ECQ_la: general linguistic usage; ECQ_hk: housekeeping; ECQ_dr: daily routines; ECQ_ms: manual skills; ECQ_m: mobility; PSQI_C1: subjective sleep quality; PSQI_C4: habitual sleep efficiency; PSQI_C5: sleep disturbances; PSQI_C6: use of sleep medications; PSQI_C7: daytime dysfunction; MMSE: Mini-Mental State Examination; ECQ_tot: Everyday Competence Questionnaire, total score.

One-way ANOVA revealed several significant group effects. Specifically, the Is group showed significantly higher scores compared to the NAc group on the depression scale (GDS—F_1,167_ = 19.567; *p* = <0.001; η^2^ = 0.101; 95% CI: 3.88–4.84) and significantly lower scores in general cognitive functioning (MMSE—F_1,141_ = 43.585; *p* = <0.001; η^2^ > 0.014; 95% CI: 24.38–25.69). Moreover, the Is group showed significantly lower scores in daily living skills and specifically in subjective well-being (ECQ_swb—F_1,166_ = 8.597; *p* = 0.004; η^2^ = 0.048; 95% CI: 2.40–2.64), linguistic abilities (ECQ_la—F_1,137_ = 23.014; *p* < 0.001; η^2^ = 0.112; 95% CI:2.67–2.81), housekeeping (ECQ_hk—F_1,127_ = 143.151; *p* < 0.001; η^2^ > 0.014; 95% CI: 4.07–5.09), daily routine (ECQ_dr—F_1,128_ = 117.677; *p* < 0.001; η^2^ > 0.014; 95% CI: 7.27–8.59), manual skills (ECQ_ms—F_1,141_ = 8.284; *p* = 0.005; η^2^ = 0.049; 95% CI: 1.75–2.36), mobility (ECQ_m—F_1,143_ = 29.761; *p* < 0.001; η^2^ > 0.014; 95% CI: 1.27–1.79), and ECQ total score (ECQ_tot—F_1,158_ = 61.084; *p* < 0.001; η^2^ > 0.014; 25.62–29.63). Finally, regarding sleep quality and complaints, the Is group showed significantly higher scores in sleep quality (PSQI_C1—F_1,71_ = 8.091; *p* = 0.006; η^2^ = 0.061; 95% CI: 0.99–1.19), subjective sleep quality (PSQI_C4—F_1,124_ = 9.028; *p* = 0.003; η^2^ = 0.133; 95% CI: 2.17–2.57), sleep disturbances (PSQI_C5—F_1,124_ = 21.648; *p* < 0.001; η^2^ = 0.097; 95% CI: 1.17–1.34), the use of sleep medications (PSQI_C6—F_1,128_ = 15.388; *p* < 0.001; η^2^ = 0.064; 95% CI: 0.46–0.84), and daytime dysfunction (PSQI_C7—F_1,103_ = 5.569; *p* = 0.02; η^2^ = 0.032; 95% CI: 0.67–0.92) subscores. These statistically significant scores are depicted in Figure 3. No statistically significant differences between the groups were seen for other ECQ (ECQ_lsa *p* = 0.157; ECQ_s *p* = 0.679) or PSQI (PSQI_C2 *p* = 0.775; PSQI_C3 *p* = 0.261; PSQI_tot *p* = 0.8) subscores.

Finally, an ANCOVA was conducted to assess the effects of demographic variables (age, education, and gender) on the variables of interest previously mentioned, and the analysis revealed no significant effects for any of the demographic factors.

## 4. Discussion

The present study aimed to investigate the impact of lifestyle and environmental context on elderly people in terms of their cognitive functioning, psychological well-being, sleep quality, and daily living skills.

Regarding lifestyle, compared with non-active individuals, the active ones exhibited lower levels of depression, fewer sleep complaints, and better daily living skills, such as participation in sports, recreational activities, and manual skills. Physical activity, whether through exercise, sports, or even daily routine, has been consistently linked with reduced symptoms of depression and sleep disturbances across various age groups and populations [35]. Physical activity is a powerful tool for improving overall psychological health [36]. Whether through structured exercise or daily motor activity, incorporating physical activity into one’s routine can lead to significant and lasting improvements in an individual’s mood and well-being. The evidence strongly supports the idea that staying active is not only beneficial for physical health but also plays a crucial role in maintaining and enhancing mental health. A recently published study [22] showed that different types of physical activity, as well as the intensity and frequency with which they are practiced, are factors able to promote an active aging process, protecting the psychophysical well-being and the general cognitive functioning of the elderly. The mechanisms linking daily life activities (including physical activity) to improved mental well-being are multifaceted: (1) biological factors, such as better neurotransmitter regulation, reduced inflammation, and increased neurogenesis; (2) psychological factors, including improved self-esteem, reduced stress, and enhanced cognitive functioning; and (3) social factors, like increased social interactions and an expanded support network [37]. A recent global estimate showed that one in four (27.5%) adults [38] and more than three-quarters (81%) of adolescents [39] do not meet the recommendations for aerobic exercise outlined in the 2010 global recommendations on physical activity for good health [40]. There is an urgent need to increase the prioritization and investment directed towards services to promote physical activity both within health and other key sectors. Our data align with the guidelines published by the WHO [36], indicating that individuals with a more active lifestyle who regularly engage in sports activities, maintain good social relationships, and pursue interests and hobbies exhibit better psychological well-being, fewer sleep disturbances, and more functional daily living skills.

Regarding environmental context, our data highlight that the institutionalized individuals showed higher levels of depression and reduced cognitive functioning, which were linked to more sleep disturbances and worse daily living skills, including participation in sports, recreational activities, subjective well-being, and manual tasks. These effects highlighted by our research were evident even though the subjects involved in the study had been living in an institutionalized setting for no more than one year. Therefore, the institutionalization of older adults, such as their placement in nursing homes or long-term care facilities, has profound implications on cognitive function, sleep quality, and independence right from the beginning. The environment and routines practiced within these settings can significantly influence these aspects of health [41]. Several studies have shown that elderly individuals in nursing homes are at higher risk of cognitive decline compared to those living in community settings [29,42]. Mechanisms contributing to cognitive and mental decline in institutionalized individuals can be multiple: social isolation, lack of cognitive and physical stimulation, environmental stressors, and inadequate healthcare.

Thus far, our data have highlighted how an active lifestyle can predispose individuals to fewer depressive symptoms, fewer sleep disturbances, and greater daily living skills. Additionally, the environmental context in which an individual resides, such as a care institution, negatively affects psychological well-being, cognitive functioning, sleep quality, and overall levels of functionality. Moreover, our results showed that these differences between groups are not influenced by demographic characteristics.

Given these findings, our third comparison aimed to determine which factor had a greater impact on an individual’s psycho-physical well-being—a care institution environmental context or an inactive lifestyle. Our data highlight that the institutionalized group showed higher levels of depression and reduced cognitive functioning, more sleep disturbances, and worse daily living skills in nearly all of the considered subscores. These data unequivocally confirm the role of the environmental context, particularly care institutions, as a major predisposing factor for developing depressive symptoms, cognitive decline, sleep disturbances, and loss of functionality in daily living activities, regardless of the demographic variables considered. The negative effects of the institutionalized care context appear to manifest even after a relatively short exposure to the context itself; in fact, all the subjects involved had been residents of the facilities for no more than one year. Promoting activity and providing cognitive and physical stimulation in care institutions for elderly individuals is crucial for maintaining their overall health, well-being, and quality of life.

In conclusion, considering both the effect sizes and the remarkable number of participants for each group, the present data seem particularly reliable and offer a good level of generalizability of results. When comparing the active and non-active groups, for example, the NAc individuals showed depression scores 1.5 greater than Ac individuals, while daily living skills were almost 25% lower. Similarly, when comparing institutionalized and non-active groups, the global cognitive functioning as assessed by the MMSE showed a difference of more than 4 points, with the mean of the institutionalized group significantly below the traditional clinical cut-off of 24 points. All these measures showed a good coincidence between them, indicating a satisfactory quality of data obtained with classical questionnaires and tests such as the GDS, MMSE, and PSQI, as well as the ECQ, used for the first time in an Italian population.

Nonetheless, the present study shows some limitations. Firstly, we were unable to evaluate cognitive and psychological functioning more comprehensively, which would have allowed us to highlight potential effects on specific and circumscribed domains such as executive functions, attention, memory, and anxiety symptoms. Secondly, although the number of subjects involved is relatively high, we could not obtain all the necessary information regarding ongoing pharmacological treatments/comorbidity and, therefore, could not assess their potential impact on the variables of interest. To mitigate the bias associated with the lack of information on pharmacotherapy/comorbidity, we only included subjects without severe motor impairments that would hinder independent walking and those without clear signs of cognitive decline.

## 5. Conclusions

This research highlights that an active lifestyle and a non-institutionalized environment, which allow for greater mobility and autonomy, are two factors that positively contribute to the mental and physical well-being of elderly individuals. Furthermore, the healthcare institution context appears to have a greater negative impact on the psycho-physical well-being of the subjects involved compared to a non-active lifestyle. Engaging in regular physical activity (e.g., jogging, going to the gym, playing tennis, etc.) or maintaining social connections through hobbies (e.g., volunteering, participating in political parties, attending the cinema/theater, etc.) can improve physical and emotional health, thereby promoting healthy aging and increasing personal independence. In this framework, an active lifestyle and a non-institutional environment contribute to successful aging and serve as protective factors against cognitive decline and psychological distress, embodying a complex strategy through which the brain strives to resist damage caused by physiological aging. Further studies should investigate the effects, for example, of an active lifestyle on specific cognitive functions (e.g., memory, executive functions, attention, etc.) and on various emotional aspects such as mood and anxiety. Additionally, it would be interesting to assess the effects of certain physical and social characteristics of environments (e.g., presence of specialized multidisciplinary staff, living alone or with other family members, having access to assistive and/or entertainment technology devices, etc.) on cognitive, emotional, and behavioral functioning.

## Figures and Tables

**Table 1:** Demographic differences in active and non-active groups.

	Active (N = 102)	Non-Active (N = 80)	*p*
Age, in years	67.19 ± 6.22	68.75 ± 6.14	0.092
Education, in years	10.8 ± 4.56	9.03 ± 4.81	**0.012**
Gender, F (%)	58 (56.8)	53 (66.2)	0.198

**Table 2:** Demographic differences in institutionalized and non-institutionalized groups.

	Non-Institutionalized (N = 154)	Institutionalized (N = 91)	*p*
Age, in years	71.72 ± 6.86	79.49 ± 7.03	**<0.001**
Education, in years	8.06 ± 3.95	8.55 ± 4.92	0.442
Gender, F (%)	86 (55.8)	56 (61.5)	0.383

**Table 3:** Demographic differences in institutionalized and non-active groups.

	Institutionalized (N = 91)	Non-Active (N = 80)	*p*
Age, in years	79.49 ± 7.03	68.75 ± 6.14	**<0.001**
Education, in years	8.55 ± 4.92	9.03 ± 4.81	0.549
Gender, F (%)	56 (61.5)	53 (66.2)	0.523

## Data Availability

The datasets generated during the current study are available from the corresponding author upon reasonable request due to ethical reasons.

## References

[1] Dinse H.R. (2006). Cortical Reorganization in the Aging Brain. Prog. Brain Res..

[2] Klenk J., Rapp K., Büchele G., Keil U., Weiland S.K. (2007). Increasing Life Expectancy in Germany: Quantitative Contributions from Changes in Age- and Disease-Specific Mortality. Eur. J. Public Health.

[3] Oeppen J., Vaupel J.W. (2002). Demography. Broken Limits to Life Expectancy. Science.

[4] Franklin N.C., Tate C.A. (2009). Lifestyle and Successful Aging: An Overview. Am. J. Lifestyle Med..

[5] Moritz I., Stein C.L., World Health Organization (1999). A Life Course Perspective of Maintaining Independence in Older Age Prepared for WHO by Inka Moritz and Claudia Stein Under the Guidance of WHO’s Ageing and Health.

[6] Crews D.E. (2003). Human Senescence: Evolutionary and Biocultural Perspectives.

[7] Mahncke H.W., Connor B.B., Appelman J., Ahsanuddin O.N., Hardy J.L., Wood R.A., Joyce N.M., Boniske T., Atkins S.M., Merzenich M.M. (2006). Memory Enhancement in Healthy Older Adults Using a Brain Plasticity-Based Training Program: A Randomized, Controlled Study. Proc. Natl. Acad. Sci. USA.

[8] Singh A.S., A Paw M.J.C., Bosscher R.J., van Mechelen W. (2006). Cross-Sectional Relationship between Physical Fitness Components and Functional Performance in Older Persons Living in Long-Term Care Facilities. BMC Geriatr..

[9] Buchner D.M., Larson E.B., Wagner E.H., Koepsell T.D., De Lateur B.J. (1996). Evidence for a Non-Linear Relationship between Leg Strength and Gait Speed. Age Ageing.

[10] Motta M., Bennati E., Ferlito L., Malaguarnera M., Motta L. (2005). Successful Aging in Centenarians: Myths and Reality. Arch. Gerontol. Geriatr..

[11] Badu-Prempeh N.B.A., Carboo A.K., Amoa A., Awuku-Aboagye E., Amoah A.A., Pekyi-Boateng P.K. (2024). Determinants of Cognitive Health in the Elderly: A Comprehensive Analysis of Demographics, Health Status, and Lifestyle Factors from NHANES. Neurol. Sci..

[12] Stern Y. (2003). The Concept of Cognitive Reserve: A Catalyst for Research. J. Clin. Exp. Neuropsychol..

[13] Nucci M., Mapelli D., Mondini S. (2012). Cognitive Reserve Index Questionnaire (CRIq): A New Instrument for Measuring Cognitive Reserve. Aging Clin. Exp. Res..

[14] Pettigrew C., Soldan A. (2019). Defining Cognitive Reserve and Implications for Cognitive Aging. Curr. Neurol. Neurosci. Rep..

[15] Shen X., Wang J., Chen J., Zhang H., Shen S., Zhao X. (2024). Relationship between Participation in Leisure Activities and the Maintenance of Successful Aging in Older Chinese Adults: A 4-Year Longitudinal Study. BMC Geriatr..

[16] Portegijs E., Lee C., Zhu X. (2023). Activity-Friendly Environments for Active Aging: The Physical, Social, and Technology Environments. Front. Public Health.

[17] Zhou J.J., Kang R., Bai X. (2022). A Meta-Analysis on the Influence of Age-Friendly Environments on Older Adults’ Physical and Mental Well-Being. Int. J. Environ. Res. Public Health.

[18] Coco M., Buscemi A., Guerrera C.S., Di Corrado D., Cavallari P., Zappalà A., Di Nuovo S., Parenti R., Maci T., Razza G. (2020). Effects of a Bout of Intense Exercise on Some Executive Functions. Int. J. Environ. Res. Public Health.

[19] Stern Y., Barnes C.A., Grady C., Jones R.N., Raz N. (2019). Brain Reserve, Cognitive Reserve, Compensation, and Maintenance: Operationalization, Validity, and Mechanisms of Cognitive Resilience. Neurobiol. Aging.

[20] Statsenko Y., Habuza T., Van Gorkom K.N., Zaki N., Almansoori T.M., Al Zahmi F., Ljubisavljevic M.R., Belghali M. (2021). Proportional Changes in Cognitive Subdomains During Normal Brain Aging. Front. Aging Neurosci..

[21] Bloomberg M., Brocklebank L., Doherty A., Hamer M., Steptoe A. (2024). Associations of Accelerometer-Measured Physical Activity, Sedentary Behaviour, and Sleep with next-Day Cognitive Performance in Older Adults: A Micro-Longitudinal Study. Int. J. Behav. Nutr. Phys. Act..

[22] D’Aurizio G., Festucci F., Di Pompeo I., Tempesta D., Curcio G. (2023). Effects of Physical Activity on Cognitive Functioning: The Role of Cognitive Reserve and Active Aging. Brain Sci..

[23] Levin O., Netz Y., Ziv G. (2017). The Beneficial Effects of Different Types of Exercise Interventions on Motor and Cognitive Functions in Older Age: A Systematic Review. Eur. Rev. Aging Phys. Act..

[24] Gleeson M., Sherrington C., Keay L. (2014). Exercise and Physical Training Improve Physical Function in Older Adults with Visual Impairments but Their Effect on Falls Is Unclear: A Systematic Review. J. Physiother..

[25] Ali N., Tian H., Thabane L., Ma J., Wu H., Zhong Q., Gao Y., Sun C., Zhu Y., Wang T. (2022). The Effects of Dual-Task Training on Cognitive and Physical Functions in Older Adults with Cognitive Impairment; A Systematic Review and Meta-Analysis. J. Prev. Alzheimers Dis..

[26] Hwang C.L., Wu Y.T., Chou C.H. (2011). Effect of Aerobic Interval Training on Exercise Capacity and Metabolic Risk Factors in People with Cardiometabolic Disorders: A Meta-Analysis. J. Cardiopulm. Rehabil. Prev..

[27] Katzmarzyk P.T., Powell K.E., Jakicic J.M., Troiano R.P., Piercy K., Tennant B. (2019). Sedentary Behavior and Health: Update from the 2018 Physical Activity Guidelines Advisory Committee. Med. Sci. Sports Exerc..

[28] Drageset J., Dysvik E., Espehaug B., Natvig G.K., Furnes B. (2015). Suffering and Mental Health among Older People Living in Nursing Homes—A Mixed-Methods Study. PeerJ.

[29] Evans I.E.M., Martyr A., Collins R., Brayne C., Clare L. (2019). Social Isolation and Cognitive Function in Later Life: A Systematic Review and Meta-Analysis. J. Alzheimers Dis..

[30] Buchman A.S., Boyle P.A., Yu L., Shah R.C., Wilson R.S., Bennett D.A. (2012). Total Daily Physical Activity and the Risk of AD and Cognitive Decline in Older Adults. Neurology.

[31] Magni E., Binetti G., Bianchetti A., Rozzini R., Trabucchi M. (1996). Mini-Mental State Examination: A Normative Study in Italian Elderly Population. Eur. J. Neurol..

[32] Yesavage J.A., Brink T.L., Rose T.L., Lum O., Huang V., Adey M., Leirer V.O. (1982). Development and Validation of a Geriatric Depression Screening Scale: A Preliminary Report. J. Psychiatr. Res..

[33] Curcio G., Tempesta D., Scarlata S., Marzano C., Moroni F., Rossini P.M., Ferrara M., De Gennaro L. (2013). Validity of the Italian Version of the Pittsburgh Sleep Quality Index (PSQI). Neurol. Sci..

[34] Kalisch T., Richter J., Lenz M., Kattenstroth J.C., Kolankowska I., Tegenthoff M., Dinse H.R. (2011). Questionnaire-Based Evaluation of Everyday Competence in Older Adults. Clin. Interv. Aging.

[35] Schuch F.B., Vancampfort D., Firth J., Rosenbaum S., Ward P.B., Silva E.S., Hallgren M., De Leon A.P., Dunn A.L., Deslandes A.C. (2018). Physical Activity and Incident Depression: A Meta-Analysis of Prospective Cohort Studies. Am. J. Psychiatry.

[36] Bull F.C., Al-Ansari S.S., Biddle S., Borodulin K., Buman M.P., Cardon G., Carty C., Chaput J.P., Chastin S., Chou R. (2020). World Health Organization 2020 Guidelines on Physical Activity and Sedentary Behaviour. Br. J. Sports Med..

[37] Moon H.Y., Praag H. (2019). van Physical Activity and Brain Plasticity. J. Exerc. Nutr. Biochem..

[38] Guthold R., Stevens G.A., Riley L.M., Bull F.C. (2018). Worldwide Trends in Insufficient Physical Activity from 2001 to 2016: A Pooled Analysis of 358 Population-Based Surveys with 1·9 Million Participants. Lancet Glob. Health.

[39] Guthold R., Stevens G.A., Riley L.M., Bull F.C. (2020). Global Trends in Insufficient Physical Activity among Adolescents: A Pooled Analysis of 298 Population-Based Surveys with 1·6 Million Participants. Lancet Child. Adolesc. Health.

[40] WHO (2010). Global Recommendations on Physical Activity for Health.

[41] Seitz D., Purandare N., Conn D. (2010). Prevalence of Psychiatric Disorders among Older Adults in Long-Term Care Homes: A Systematic Review. Int. Psychogeriatr..

[42] Wilson R.S., Krueger K.R., Arnold S.E., Schneider J.A., Kelly J.F., Barnes L.L., Tang Y., Bennett D.A. (2007). Loneliness and Risk of Alzheimer Disease. Arch. Gen. Psychiatry.

